# Identifying multimorbidity profiles associated with COVID-19 severity in chronic patients using network analysis in the PRECOVID Study

**DOI:** 10.1038/s41598-022-06838-9

**Published:** 2022-02-18

**Authors:** Jonás Carmona-Pírez, Antonio Gimeno-Miguel, Kevin Bliek-Bueno, Beatriz Poblador-Plou, Jesús Díez-Manglano, Ignatios Ioakeim-Skoufa, Francisca González-Rubio, Antonio Poncel-Falcó, Alexandra Prados-Torres, Luis A. Gimeno-Feliu, on behalf of the PRECOVID Group, Aida Moreno-Juste, Mabel Cano-del-Pozo, Ana Cristina Bandrés-Liso, Victoria Pico-Soler, Mercedes Aza-Pascual-Salcedo, Paula Ara-Bardají

**Affiliations:** 1 EpiChron Research Group, Aragon Health Sciences Institute (IACS), IIS Aragón, Miguel Servet University Hospital, Zaragoza, Spain; 2 Health Services Research On Chronic Patients Network (REDISSEC), Network for Research On Chronicity, Primary Care, and Health Promotion (RICAPPS), ISCIII, Madrid, Spain; 3 Delicias-Sur Primary Care Health Centre, Aragon Health Service (SALUD), Zaragoza, Spain; 4 Preventive Medicine and Public Health Teaching Unit, Miguel Servet University Hospital, Zaragoza, Spain; 5 Internal Medicine Department, Royo Villanova Hospital, Zaragoza, Spain; 6 WHO Collaborating Centre for Drug Statistics Methodology, Norwegian Institute of Public Health, NO-0213 Oslo, Norway; 7 Department of Drug Statistics, Division of Health Data and Digitalisation, Norwegian Institute of Public Health, NO-0213 Oslo, Norway; 8 Drug Utilization Work Group, Spanish Society of Family and Community Medicine (SEMFYC), S08009 Barcelona, Spain; 9 Aragon Health Service (SALUD), 50017 Zaragoza, Spain; 10 San Pablo Primary Care Health Centre, Aragon Health Service (SALUD), University of Zaragoza, Zaragoza, Spain; 11 Aragon Health Service, Zaragoza, Spain; 12 Department of Health, Government of Aragon, Zaragoza, Spain; 13 Aragon Health Service (SALUD), Zaragoza, Spain; 14 Torrero-La Paz Primary Care Health Centre, Aragon Health Service (SALUD), Zaragoza, Spain; 15 Primary Care Pharmacy Service Zaragoza III, Aragon Health Service (SALUD), Zaragoza, Spain; 16 Aragon Institute for Health Research (IIS Aragón), Aragon, Spain

**Keywords:** Public health, Epidemiology, Statistics, Health care, Risk factors

## Abstract

A major risk factor of COVID-19 severity is the patient's health status at the time of the infection. Numerous studies focused on specific chronic diseases and identified conditions, mainly cardiovascular ones, associated with poor prognosis. However, chronic diseases tend to cluster into patterns, each with its particular repercussions on the clinical outcome of infected patients. Network analysis in our population revealed that not all cardiovascular patterns have the same risk of COVID-19 hospitalization or mortality and that this risk depends on the pattern of multimorbidity, besides age and sex. We evidenced that negative outcomes were strongly related to patterns in which diabetes and obesity stood out in older women and men, respectively. In younger adults, anxiety was another disease that increased the risk of severity, most notably when combined with menstrual disorders in women or atopic dermatitis in men. These results have relevant implications for organizational, preventive, and clinical actions to help meet the needs of COVID-19 patients.

## Introduction

Immediately after the coronavirus disease (COVID-19) outbreak in December 2019, the clinical and research communities worldwide set their focus on identifying aspects that influence the disparity of symptoms and outcomes in infected individuals. More than a year and a half later, and even though several factors related to poor prognosis have already been identified, the ongoing COVID-19 pandemic still represents a challenge for clinicians, healthcare systems and society. Severe outcomes and complications can occur at any age, even in previously healthy individuals^1^. Nevertheless, we now know that male sex is a risk factor for poor prognosis^2–4^ and that older patients, especially those over the age of 80, are more likely to develop more severe outcomes^5,6^.

The presence of certain chronic diseases at the time of infection seems to be another determining factor of COVID-19 severity. Cardiovascular and metabolic diseases such as obesity, heart failure, chronic renal failure, and diabetes mellitus^1,7^ have been consistently associated with a higher risk of severity, while the role of other conditions such as asthma remains unclear^8^. To date, most studies on comorbidity and COVID-19 severity have analyzed the impact of some diseases individually. However, multimorbidity (i.e., the presence of two or more chronic diseases) is the norm rather than the exception in chronic patients, especially in the elderly^9^. It is well known that chronic diseases tend to cluster into non-random patterns, usually referred to as multimorbidity patterns^10^. It is expected that some patterns could affect COVID-19 prognosis in a more significant way than others, depending on the disease composition of each pattern and also on various factors such as sex and age. In this sense, some studies have already suggested that patterns linked to low-grade systemic inflammation could be decisive in COVID-19 prognosis^11^, though only a few have focused on identifying disease patterns with an impact on COVID-19 patients^12–15^.

The continuously changing landscape of the COVID-19 pandemic with the appearance of new variants of the virus with high transmission rates and unpredictable severity calls scientists to identify vulnerable chronic patients. Characterization of the clinical profiles of the population at higher risk of hospitalization or mortality may help us effectively design and develop preventive strategies targeting people with specific chronic diseases (for both primary and secondary prevention). It can also give us valuable information to better understand the etiopathological characteristics of the infection and its complications, and potentially generate hypotheses for a more appropriate clinical management of patients with multiple chronic conditions. The use of electronic health records and COVID-19 registries can bring potential improvements in population health, increasing research efficiency and data quality and management^16,17^, among other benefits. Real-world data can generate solid scientific evidence for important questions that challenge healthcare professionals and public health systems. In this context, innovative data-mapping tools, such as network analysis, could help detect patterns from within the wide range of conditions and their potential combinations, discriminating between different high-risk profiles of infected individuals based on their underlying comorbidities. Network analysis applies community detection methods and has been used to study multimorbidity patterns through the correlations among diseases^18,19^. However, this is the first time to the extent of our knowledge that this method is applied to study the correlations between individuals based on common comorbidities, and more specifically in COVID-19 patients, in order to detect higher-risk severity profiles.

This population-based study aims to (1) identify clinical profiles of COVID-19 patients based on their baseline morbidity using network analysis, (2) clinically describe the multimorbidity patterns obtained, and (3) assess their impact on infection severity (compared with chronic patients without multimorbidity).

## Results

### Characteristics of the study population: 48,415 patients, 80% had multimorbidity

The study population comprised all patients aged 15 years or older infected and with chronic diseases from June 15, 2020, to December 19, 2020, from the Spanish region of Aragon. A total of 48,415 individuals (mean age 52.0 years) with laboratory-confirmed COVID-19 infection (approximately 4% of the reference population) were included. Eight in every ten of them had multimorbidity, with a mean number of 3.9 chronic diseases per patient (Table 1), slightly higher in women than in men. During follow-up, 9.6% of the individuals developed severe infection; of those, 9.5% were admitted to the hospital, and 2.7% died.

**Table 1:** Demographic and clinical characteristics of individuals aged 15 years or older with COVID-19 infection in the PRECOVID Cohort.

Characteristics	Total (N = 48,415)	Men (N = 20,936)	Women (N = 27,479)
Mean age (sd)	52.0 (21.10)	51.7 (20.47)	52.3 (21.56)
**Age interval (N, %)**			
15–64 years	35,106 (72.5%)	15,279 (73.0%)	19,827 (72.2%)
65–79 years	6,961 (14.4%)	3,431 (16.4%)	3,530 (12.8%)
≥ 80 years	6,348 (13.1%)	2,226 (10.6%)	4,122 (15.0%)
Mean number of chronic diseases (sd)	3.94 (2.88)	3.50 (2.61)	4.29 (3.02)
**Multimorbidity † (N, %)**			
Total	38,323 (79.2%)	15,659 (74.8%)	22,664 (82.5%)
15–64 years	25,605 (72.9%)	10,306 (67.5%)	15,299 (77.2%)
65–79 years	6,568 (94.4%)	3,190 (93.0%)	3,378 (95.7%)
≥ 80 years	6,150 (96.9%)	2,163 (97.2%)	3,987 (96.7%)
Outcomes			
Hospitalization (N, %)	4,586 (9.5%)	2,400 (11.5%)	2,186 (8.0%)
30-day-mortality (N, %)	1,311 (2.7%)	683 (3.3%)	628 (2.3%)
**Severity †† (N, %)**			
Total	5,117 (10.6%)	2,671 (12.8%)	2,446 (8.9%)
15–64 years	1,572 (4.5%)	903 (5.9%)	669 (3.4%)
65–79 years	1,338 (19.2%)	790 (23.0%)	548 (15.5%)
≥ 80 years	2,207 (34.8%)	978 (43.9%)	1,229 (29.8%)

^†^ Multimorbidity, defined as the simultaneous co-existence of two or more chronic diseases, excluding COVID-19; sd, standard deviation; **††** Severity, defined as a composite outcome based on the need for hospital admission (including in Intensive Care Units) or 30-day all-cause mortality.

**Figure 1:**
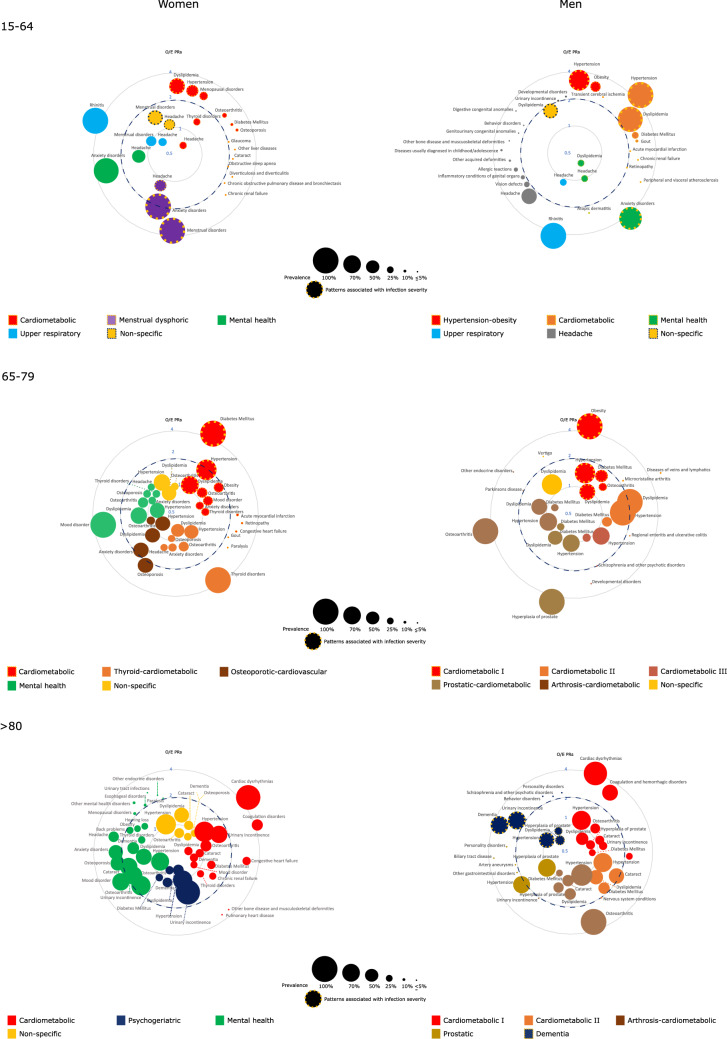
Disease composition of the multimorbidity patterns identified in the study population based on sex and age, accordign to prevalence (proportional to node size) and observed/expected prevalence ratios (O/E PRs). A disease was included as a part of a pattern when presented a prevalence greater than 25% and/or a O/E PR ≥ 2 (witha prevalence of at least 1%). In broken lines patterns with statistically significant age-adjusted odds ratios of infection severity.

The most frequently observed chronic diseases/conditions were cardio-metabolic (i.e., hypertension, dyslipidemia, diabetes, and obesity), cardiovascular (i.e., ischemic heart disease, cardiac dysrhythmia, and heart failure), chronic respiratory (i.e., asthma, rhinitis, chronic obstructive pulmonary disease -COPD-, and obstructive sleep apnea-OSA), and mental health disorders (i.e., anxiety and mood disorders) (Fig. 1). In the 38,823 patients with two or more chronic diseases included in the network analysis, these conditions were combined into up to fourteen different multimorbidity patterns with certain sex- and age-specificities and a differential impact on COVID-19 severity (Table 2). The complete output of the pattern analysis is available as supplementary material in which we detailed the complete disease analysis with their prevalence and observed/expected prevalence ratios.

**Table 2:** Denomination and prevalence of multimorbidity patterns found in COVID-19 patients based on sex and age and their age-adjusted odds ratios (aOR) of infection severity compared with chronic patients without multimorbidity.

	15–64 years		65–79 years		≥ 80 years	
	Pattern name (N, prevalence %)	Severity aOR (95% CI)	Pattern name (N, prevalence %)	Severity aOR (95% CI)	Pattern name (N, prevalence %)	Severity aOR (95% CI)
**Women**	**Cardiometabolic (4,205, 21.4%)**	**1.85 (1.43–2.41)**	**Cardiometabolic (673, 19.1%) (99% diabetes mellitus)**	**1.97 (1.15–3.49)**	Cardiometabolic (541, 13.1%)	1.29 (0.85–1.96)
	**Menstrual-dysphoric (2,204, 11.1%)**	**1.48 (1.08–2.02)**	Thyroid-cardiometabolic (515, 14.6%) (no diabetes mellitus)	1.55 (0.86–2.79)	Psychogeriatric (1,011, 24.5%)	1.06 (0.71–1.58)
	Mental health (2,046, 10.3%)	0.90 (0.63–1.29)	Osteoporotic-cardiovascular (70, 19.9%) (no diabetes mellitus)	1.20 (0.67–2.14)	Mental health (753, 18.3%)	1.32 (0.87–1.98)
	Upper respiratory (1,987, 10.0%)	1.13 (0.79–1.62)	Mental health (823, 23.3%) (no diabetes mellitus)	1.58 (0.90–2.80)	Non-specific (1,679, 40.8%)	1.05 (0.71–1.55)
	**Non-specific (4,810, 24.3%)**	**1.35 (1.02–1.77)**	Non-specific (663, 18.8%) (no diabetes mellitus)	1.22 (0.68.2.18)		
	Control group (4,528, 22.8%)		Control group (152, 4.3%)		Control group (135, 3.3%)	
**Men**	**Hypertension-obesity (1,585, 10.4%)**	**1.60 (1.25–2.05)**	**Cardiometabolic I (421, 12.3%) (99% obesity)**	**1.76 (1.18–2.62)**	Cardiometabolic I (546, 24.6%)	1.28 (0.96–1.70)
	**Cardiometabolic (1,136, 7.4%)**	**1.43 (1.14–1.80)**	Cardiometabolic II (619, 18.1%) (no obesity)	1.23 (0.84–1.81)	Cardiometabolic II (590, 26.5%)	1.28 (0.96–1.70)
	**Mental Health (1,911, 12.5%)**	**1.35 (1.06–1.74)**	Cardiometabolic III (637, 18.6%) (no obesity)	1.41 (0.96–2.06)	Arthrosis-cardiometabolic (444, 20.0%)	1.28 (0.96–1.71)
	Upper respiratory (1,446, 9.5%)	1.30 (0.97–1.75)	Prostatic-cardiometabolic (699, 20.4%) (18% obesity)	1.30 (0.89–1.89)	Prostatic (233, 10.5%)	1.30 (0.92–1.84)
	Headache (1,949, 12.8%)	1.24 (0.93–1.64)	Arthrosis-cardiometabolic (394, 11.5%) (no obesity)	1.20 (0.80–1.82)	**Dementia (348, 15.6%)**	**1.42 (1.04–1.94)**
	**Non-specific (2,274, 14.9%)**	**1.18 (1.03–1.60)**	Non-specific (416, 12.1%) (no obesity)	1.28 (0.85–1.93)		
	Control group (4,973, 32.6%)		Control group (241, 7.0%)		Control group (63, 2.8%)	

In bold letters, patterns with statistically significant age-adjusted odds ratios of infection severity; Control group: chronic patients without multimorbidity; CI: confidence interval.

### Multimorbidity patterns in women and impact on infection severity: menstrual-dysphoric and cardiometabolic patterns highlight

We identified eight multimorbidity patterns in women, which were classified as cardiometabolic, thyroid-cardiometabolic, osteoporotic-cardiovascular, upper respiratory, mental health, psychogeriatric, menstrual-dysphoric, and non-specific. Their disease composition, specificities according to age, and impact on infection severity are described below and in Fig. 1 and Table 2.

In women aged 15–64, we found five patterns. The cardiometabolic pattern, which included 15 diseases such as hypertension, dyslipidemia, diabetes, and menopausal disorders amongst others, was associated with an 86% increase in severity (age-adjusted odds ratio OR 1.86, 95% confidence interval CI 1.43–2.41) compared with individuals with only one chronic condition. The menstrual-dysphoric pattern included anxiety, menstrual disorders, and headaches, and associated (OR [95% CI]) the second highest severity risk (1.48 [1.08–2.02]). A less-specific pattern with menstrual disorders as its most prevalent chronic conditions was also described to increase severity by 35% (1.35 [1.02–1.77]). The two remaining patterns were an upper respiratory pattern and a mental health pattern, none of which was associated with higher COVID-19 infection severity compared to the reference group that included chronic patients without multimorbidity.

Five other patterns were identified in women aged 65–79 years. An advanced cardiometabolic pattern including 13 diseases such as diabetes (present in 99% of women), obesity, hypertension, retinopathy, heart failure, acute myocardial infarction, and gout, was associated (OR [95% CI]) with severity (1.97 [1.12–3.49]). Four other patterns in which the prevalence of diabetes did not exceed 1% and tagged as thyroid-cardiometabolic, osteoporotic-cardiovascular, mental health, and non-specific, did not associate higher severity risk.

In women aged 80 and older, we identified four multimorbidity patterns, none of which associated higher severity risk compared with the chronic reference group. Most cases were grouped into a non-specific pattern. The cardiometabolic pattern in this group included cardiac dysrhythmias, coagulation/ hemorrhagic disorders, heart failure, and acute myocardial infarction, while the mental health pattern included mood disorders, anxiety, and cognitive disorders. We also found a psychogeriatric group with urinary incontinence and dementia.

### Multimorbidity patterns in men and impact on infection severity: mental health, cardiometabolic and dementia patterns highlight

A total of ten multimorbidity patterns were identified in men, which we referred to as cardiometabolic, hypertension-obesity, arthrosis-cardiometabolic, prostatic-cardiometabolic, prostatic, mental health, upper respiratory, headache, dementia, and non-specific. Their composition, age-specificities and effects on COVID-19 severity are described below and in Fig. 1.

In men aged 15–64 years, six patterns were identified. A hypertension-obesity pattern including hypertension, obesity, and transient cerebral ischemia was associated (OR [95% CI]) the most with infection severity (1.60 [1.25–2.05]). We also found a cardiometabolic group, mainly including hypertension and dyslipidemia, was also associated with a 43% increase in severity (1.43 [1.14–1.80]). A mental health group with anxiety disorders and atopic dermatitis was also associated with a 36% higher risk of severity (1.36 [1.06–1.74]). The non-specific pattern, with dyslipidemia as the most prevalent chronic condition, was also described to increase severity by 28% (1.28 [1.03–1.60]), in addition to two other patterns unrelated to severity named (upper-tract) respiratory pattern and headache pattern.

Six multimorbidity patterns were described in men aged 65–79. The only one associated with higher severity risk (OR [95% CI]) was a cardiometabolic pattern (1.76 [1.18–2.62]) that included seven diseases: obesity (present in 99% of men), hypertension, dyslipidemia, diabetes, osteoarthritis, microcrystalline arthritis, and diseases of veins and lymphatics. Five other patterns unrelated to severity were also described: two other presentations of the cardiometabolic pattern (cardiometabolic II and III), two patterns also with these kinds of conditions (prostatic-cardiometabolic and arthrosis-cardiometabolic), and a non-specific multimorbidity pattern. In these groups, the prevalence of obesity did not exceed 1%, except in the prostatic-cardiometabolic one (18%).

In men aged 80 years and older, we detected five patterns. A psychogeriatric pattern comprising urinary incontinence, cognitive disorders, and behavioral and mental disorders among other diseases, was the only one associated (OR [95% CI]) with increased infection severity risk (1.42 [1.04–1.94]). The four other patterns unrelated to severity were defined as prostatic, cardiometabolic I, cardiometabolic II, and arthrosis-cardiometabolic.

## Discussion

In this large-scale study, we explored network analysis as a new approach to identify the characteristics behind different profiles of COVID-19 individuals that could explain the disparities observed in infection severity based on their underlying morbidity. Our results evidenced the presence of up to fourteen multimorbidity patterns, most of which included cardiometabolic and vascular diseases as the most frequent chronic conditions, with varying effects on infection severity depending on sex, age, and comorbidity. Our findings can contribute to the improvement of the healthcare organization of COVID-19 patients and help direct health interventions and shielding strategies towards the most vulnerable chronic patients.

Our study revealed that multimorbidity patterns comprising diseases of predominantly cardiometabolic nature were the ones most consistently associated with infection severity. These findings align with previous studies that have identified cardiometabolic (i.e., diabetes, obesity, hypertension, dyslipidemia)^7^ and cardiorespiratory diseases (i.e., ischemic heart disease, cardiac dysrhythmia, heart failure, COPD, OSA)^1^ as severity risk factors. However, not all cardiometabolic patterns seemed to be related with increased severity in our population.; obesity in men and diabetes in women were differential diseases in high-risk cardiometabolic patterns, which supports the low-grade systemic inflammation pattern hypothesis in COVID-19^11^. This study identified combinations of diseases responsible for the most severe cases of infection that should be considered in effective prevention strategies. Below we discuss such combinations considering sex and age group, focusing on the most severe and novel ones.

Cardiometabolic patterns were strongly associated with a high risk of severity. In patients aged 65–79, we observed important sex differences in the composition of the cardiometabolic pattern: diabetes was the differential disease in women, present in 99% of patients, while obesity was in men, present in 99% of individuals. Surprisingly, although these two diseases are very common in the general population, we observed a low prevalence (below 1%) in the rest of the patterns identified in this age group in both sexes. This suggests that diabetes in women and obesity in men could act as differential factors or triggers that attract other diseases in this stratum and configure the most severe patterns in both men and women.

A medical history of metabolic and vascular diseases is a risk factor for additional comorbidity and potential complications. Many of those patients can present a cardiometabolic and/or respiratory pattern. Thus, underlying chronic conditions must be taken into consideration for the clinical management of infected patients. Severity, in general, seems to be linked to more advanced stages of the cardiometabolic pattern, which include diseases such as COPD, OSA, heart failure, chronic renal failure, or retinopathy, where target organs can sustain significant damage over time.

Low-grade chronic systemic inflammation is characterized by a proinflammatory state with increased macrophage infiltration in peripheral cells. Such state does not involve loss of function but is strongly related to the appearance of multiple cardiovascular diseases. It starts with an inflammation of the visceral adipose tissue that leads to insulin resistance, connecting obesity, diabetes, and cardiovascular diseases^20^. The results of our work are consistent with previous studies highlighting the importance of chronic systemic inflammation on COVID-19 severity^11^. The association between chronic inflammation status and infection severity has also been described at molecular level, especially in aging^21,22^. The existence of low levels of HMGA1 gene may play a key role as a risk factor for COVID-19 patients by triggering inflammatory pathways and atherosclerosis^22,23^. Low levels of HMGA1 in airway tissues are also associated with smoking and COPD^24^, connecting cardio and respiratory diseases with COVID-19 severity.

In women, patterns including menstrual and menopausal disorders were associated with higher infection severity. Menopausal disorders were part of an advanced cardiometabolic pattern that had hypertension and dyslipidemia at its core, but which also included conditions related to organ failure such as heart failure, chronic renal failure or COPD.

In young and adult population of up to 64 years of age, patterns with anxiety disorders were associated with severity, in men with atopic dermatitis and in women with menstrual disorders. The role of chronic anxiety in severity risk is still unclear. It seems that anxiety affects general health, but can also have an impact on protective factors for COVID-19 perceived risk such as the attitudes towards relationships or self-esteem^25^. A chronically anxious population with even higher anxiety levels would deteriorate general health and protective factors, including risk perception. The dysphoric-menstrual pattern and its impact on severity also deserve further investigation, not just related to anxiety, but also to understand its connection with menstrual disorders, which has not been related to severity risk before.

Age is one of the most influencing factors in COVID-19 prognosis^26^. In advanced ages, it seems that the effect of multimorbidity on severity is, in general, not significant when compared with the presence of just one chronic condition. A dementia pattern in men was the only one associated with severity in the population of 65 years of age and over. Previous studies have also linked dementia, male sex, and age with higher clinical severity^27^. Patients with dementia have multiple comorbidities such as diabetes and pneumonia, higher baseline inflammation and limited capacity to follow protective recommendations, factors that could explain this result^28^. Our results are consistent with the importance of age and sex in COVID-19 infection^3,4,22,29^; the study of such repeatable and straightforward variables that are accessible to health professionals and policy-makers could help to develop sex-balance and age-adaptive guidelines^29^.

One of the main strengths of our study is its large-scale population-based nature, including all individuals with laboratory-confirmed infection in the region. In relation to this real-world dataset, another strength of this study is its innovative approach to detect communities of COVID-19 patients based on their multimorbidity patterns using network analysis. This is the first time to the extent of our knowledge that this methodology is applied for this purpose. Network science studies the collective behavior of interconnected elements and how patterns emerge from them. It has developed several tools to visualize and exhaustively analyze big real-world data sets to predict their behavior and improve the objectivity of the analysis. Network analysis and this approach in particular facilitates the automation and replicability of this analysis compared to other classical cluster techniques that allow analyzing patients as the grouping unit, but present relevant computational limitations (e.g., agglomerative hierarchical methods) or higher subjectivity (e.g., k-means clustering)^30^. This paper shows the potential to apply this method to the study of patient associations based on their common underlying chronic conditions. In connection to this network approach, another principal strength of our research is that we exhaustively analyzed virtually all chronic diseases (operationalized into 153 chronic conditions) obtained from primary sources of information (i.e., patients' electronic health records -EHRs), and not only those most prevalent, relevant, or self-reported by the patients. The most important limitation is that our database only included information on all-cause mortality and not on the cause of death, so we could not assess the direct association between COVID-19 infection and death. On the other hand, some variables that were not available in our cohort could have been relevant in the interpretation of the results, such as socio-economic variables, genetics, laboratory tests, and inpatient treatments, amongst others.

## Conclusions

In conclusion, network analysis could help us to discern which specific combinations of chronic diseases are behind most severe cases of COVID-19 infection. Its application to our large-scale population-based cohort reveals the presence of multimorbidity patterns with a differential impact on infection severity based on age, sex, and disease composition. Our results support the importance of cardiovascular and metabolic disease patterns as aggravating factors of the infection as well as other disease combinations, mainly including anxiety, which had not been described as risk factors, thus deserving future investigation. We hypothesize that diabetes mellitus in women and obesity in men play an important role as disease attractors within their respective sex-specific multimorbidity patterns, which are associated with severity in patients over the age of 65 years. This new approach could be especially relevant for young-adult individuals in which different multimorbidity patterns are associated with increased severity risk, whereas age seems to be the most influencing factor of severity in older patients, regardless of their type of multimorbidity. Our findings can be helpful in the identification of at-risk chronic patients that should be the priority targets of shielding and close monitoring strategies during the pandemic.

## Methods

### Design and study population

We performed a retrospective, observational study in the PRECOVID cohort^31^, which includes demographic and clinical information of all the users of the public health system with laboratory-confirmed infection by SARS-CoV-2 in Aragon, a region of northeastern Spain with a reference population of 1.3 million inhabitants. Aragon's Health System provides universal health coverage for all citizens at no cost, and is used by approximately 98% of the reference population in the region^32^. For this study, we included all 48,415 individuals aged 15 years or older, infected, and with chronic diseases from June 15, 2020 to December 19, 2020. We excluded patients below the age of 15 years due to the low prevalence of chronic conditions, multimorbidity and severe COVID-19 cases in this group. Patients were followed for a maximum of 30 days from the index date (i.e., date in which the confirmatory test sample was taken) or until the date of hospitalization and/or death within this period to analyze infection severity. Severity was measured as a composite outcome based on the need for hospital admission (including in Intensive Care Units) or 30-day all-cause mortality. We operationalized severity in this way to distinguish between mild and moderate-to-severe cases.

The Clinical Research Ethics Committee of Aragón (CEICA) approved the research protocol for this study (PI20/226). CEICA waived the requirement to obtain informed consent from the participants included in this study due to its epidemiological nature and the use of anonymized data. We performed this study following the Declaration of Helsinki and the Spanish Law on the protection of personal data (LOPD 15/1999 of December 14).

### Study variables and data sources

For each individual, we analyzed sex, age (15–64, 65–79, ≥ 80 years), and all baseline chronic diseases from patients' EHRs present at the time of inclusion in the cohort. Diagnoses were classified using the International Classification of Primary Care, First Edition (ICPC-1), which was later mapped to the International Classification of Diseases, 9th revision, Clinical Modification (ICD-9-CM) codes^33^. These codes were then assigned to 226 clinical categories based on the Clinical Classification Software^34^, 153 of which were classified as chronic according to the Chronic Condition Indicator software^35^. The software considers as such those present at least during the last 12 months and that meet one or both of the following criteria: (a) entail limitations on self-care, independent living, and social interactions; (b) require of ongoing interventions using medical products, services, and special equipment. To facilitate their clinical interpretation, some diagnostic labels were renamed by the clinicians of the group. Multimorbidity was defined as the presence of two or more chronic conditions meeting the aforementioned criteria.

During follow-up, we analyzed patient hospitalization (including admission to the ICU) and mortality. Patients were followed for a maximum of 30 days, considering difficult to attribute to COVID-19 infection any events occurring afterwards, seeing as the exact cause of death was not available. Regarding hospital admissions, only those occurring within 15 days of the index date were considered a consequence of the infection. Since some patients were diagnosed only after hospitalization, we also accounted for those occurring up to 15 days before the index date.

The study variables were obtained from patient EHRs, the user's health database, and an ad hoc COVID-19 registry developed by the Aragonese Health System that links all data at a person level and in a pseudo-anonymized form.

### Statistical analysis

This multistep study aims to (1) identify clinical profiles of COVID-19 patients based on their baseline morbidity using network analysis, (2) to clinically describe the multimorbidity patterns obtained, and (3) to assess their impact on infection severity (compared with chronic patients without multimorbidity).

First, we described the population's demographic and clinical characteristics as means and/or frequencies.

Then, to describe the multimorbidity patterns, we first applied network analysis in the population with multimorbidity, stratifying by sex and age, to identify communities of similar patients in each subpopulation based on all their chronic conditions. Network science studies maps of linked components to understand complex systems, capturing that it is challenging to infer their collective behavior based on the knowledge of the system's elements^36^.

Due to the dichotomous nature of the diagnostic variables (i.e., presence/absence), the Jaccard index (JI) was used to measure the similarity between patients, just as in previous multimorbidity pattern studies^30,37^. This coefficient measures the distance between patients based on individual and shared characteristics (chronic conditions in our case), ignoring diagnoses that none of them has. Its formula is the intersection over the union of the datasets analyzed, and we applied it to each pair of patients based on their disease datasets. A JI = 1 means that two individuals are identical, and a JI = 0 means that they share no values. We considered a link between patients if the JI between them was ≥ 0.33 to analyze patients who share with another at least half of their chronic conditions. Thus, a node in each network represents a different patient, and a link means a JI ≥ 0.33 between patients. Patients aged 15–64 had, on average, four diseases, so if two patients have four diseases each and share two of them, their JI is 0.33 (intersection/union = 2/6 = 0.33). This threshold is particularly determinant in patients with two diseases, a very frequent case, especially in young patients; if these individuals share one disease, their JI is 0.33 (1/3 = 0.33). A higher threshold deletes them unless they have an identical pair or share the same diseases with other patients, which is rare, hyper-fragmenting the networks and creating tiny communities. The stratum aged 65–79 had six chronic conditions on average; two patients with such diseases pass the threshold only if they share at least three of them (3/9 = 0.33). Patients aged 80 and older had, on average, seven diseases, and two patients with such characteristics have to share at least four diseases (4/10 = 0.4) to pass the threshold.

This cut-off for building the patient networks allowed for the inclusion of almost all the patients with multimorbidity (38,303 out of 38,323 patients). At the same time, it only included 7.58% of all possible combinations between patients (14,501,518 out of 191,198,218 possible combinations), saving computation memory.

The network's modularity was used to search for communities of patients within each network using the Louvain method^38^, as previously used in comorbidity and multimorbidity pattern studies^18,19^. Modularity calculates the density of links inside communities compared to the links between them^38^. By optimizing modularity in an iterated procedure, we detected communities, also called groups or clusters of patients, until modularity ceased to increase^39^. Community detection methods like this one allow the number and size of the groups to be determined by the network's structure^40^ based on the number of links between individuals and their weight (measured by the JI) and not by the researcher. This step allowed us to assign each individual to a community or cluster of patients.

Once the groups of patients were identified for each subpopulation, and in order to characterize multimorbidity patterns within each group, we calculated the prevalence of each chronic condition together with their observed/expected (O/E) prevalence ratio (i.e., the disease prevalence observed in a specific group divided by the disease prevalence observed in the subpopulation of reference). A chronic condition was included in a pattern if (1) the prevalence was higher than 25%; or (2) the O/E prevalence ratio was ≥ 2^41,42^ and the prevalence was higher than 1%. The patterns were then named by all clinicians by consensus, taking into consideration the most relevant diseases within each pattern according to their prevalence and O/E prevalence ratio, and in line with the denominations used in the literature.

Finally, to analyze the impact on infection severity of each cluster of patients characterized by a multimorbidity pattern, we performed age-adjusted logistic regression models in each subpopulation. In doing so, we used the group of patients with one chronic condition as the reference group, and calculated age-adjusted ORs accompanied by their respective 95% CI, which represented the likelihood of infection severity for each multimorbidity pattern compared with having only one chronic disease.

All the analyses were conducted using RStudio software (version 1.4.1106, RStudio, Boston, MA, US) and STATA software (Version 14.0, StataCorp LLC, College Station, TX, US).

## Supplementary Information

Supplementary Information.

## Data Availability

The data used in this study cannot be publicly shared, because of restrictions imposed by the Aragon Health Sciences Institute (IACS) and asserted by the Clinical Research Ethics Committee of Aragon (CEICA, ceica@aragon.es). The authors who accessed the data belong to the EpiChron Research Group of IACS, and received permission from IACS to utilize the data for this specific study, thus implying its exclusive use by the researchers appearing in the project protocol approved by CEICA. The EpiChron Group can establish future collaborations with other groups based on the same data. However, each new project based on these data has to be previously submitted to the CEICA to obtain the respective mandatory approval. Potential collaborations should be addressed to the Principal Investigator of the EpiChron Research Group, Alexandra Prados-Torres at sprados.iacs@aragon.es.
